# Insights into coeliac disease diagnosis: a 2021–2023 overview of diagnostic approach and delays in children in Slovenia

**DOI:** 10.1007/s10354-024-01045-9

**Published:** 2024-06-05

**Authors:** Petra Rižnik, Tina Kamhi Trop, Martina Klemenak, Tomaž Krenčnik, Tanja Milanič-Koron, Eva Miler Mojškerc, Tatjana Pavlin, Tina Požek Šavs, Janez Zupančič, Jernej Dolinšek

**Affiliations:** 1 Paediatric Department, Gastroenterology, Hepatology and Nutrition Unit, University Medical Centre Maribor, Ljubljanska ulica 5, Maribor, Slovenia; 2 Clinical Department of Paediatric Gastroenterology, Hepatology and Nutrition, University Children’s Hospital Ljubljana, Ljubljana, Slovenia; 3 Paediatric Department, General Hospital Nova Gorica, Nova Gorica, Slovenia; 4 Paediatric Department, General Hospital Slovenj Gradec, Slovenj Gradec, Slovenia; 5 Paediatric Department, General Hospital Novo mesto, Novo mesto, Slovenia; 6 Paediatric Department, General Hospital Jesenice, Jesenice, Slovenia; 7 Paediatric Department, General Hospital Celje, Celje, Slovenia; 8 Faculty of Medicine, Paediatric Department, University of Maribor, Maribor, Slovenia

**Keywords:** Coeliac disease, Paediatrics, No-biopsy approach, Duodenal biopsy, Diagnostic delays

## Abstract

**Introduction:**

Over the past decade, the European Society for Paediatric Gastroenterology, Hepatology and Nutrition (ESPGHAN) proposed the option of diagnosing coeliac disease (CD) in children without duodenal biopsy. The aim of our study was to assess the diagnostic approach in newly diagnosed children with CD in Slovenia.

**Methods:**

In this prospective study, Slovenian paediatric gastroenterologists were invited to provide medical records of children under 19 years diagnosed with CD from March 2021 to October 2023. The analysis focused on tissue transglutaminase antibody (TGA) levels at diagnosis, diagnostic approach, adherence to ESPGHAN CD guidelines and diagnostic delays.

**Results:**

Data from 160 newly diagnosed CD patients (61.9% female; median age 8 years; 16.9% asymptomatic) were available for the analysis. No-biopsy approach was used in 65% (*N* = 104) of children and the majority (*N* = 101) fulfilled all the criteria for the no-biopsy approach. Of 56 children diagnosed using duodenal biopsy, a further 10 (17.8%) would have also been eligible for the no-biopsy approach based on the very high levels of TGA. Median diagnostic delay from first symptoms to confirmation of diagnosis was 6 months (min 0 months, max 87 months). Use of the no-biopsy approach has risen significantly since 2016 (37.8% vs. 65.0%; *p* = 0.001) and diagnostic delays have shortened (6 vs. 7 months; *p* < 0.05).

**Conclusion:**

This prospective study highlights the frequent use of a no-biopsy approach for diagnosing CD in children in Slovenia, showing large adherence to ESPGHAN guidelines. Also, diagnostic delays have shortened over recent years, likely due to various awareness-raising projects on CD conducted during this period.

## Introduction

Coeliac disease (CD) is a lifelong systemic autoimmune disorder elicited by gluten and related prolamins in genetically susceptible individuals [1]. It might present with a wide variety of gastrointestinal and extraintestinal symptoms that contribute to a significant delay in disease diagnosis, especially in adults [2]. Histological findings of villous atrophy and crypt hyperplasia with increased levels of intraepithelial T lymphocytes from duodenal biopsies, classified according to Marsh–Oberhuber, have been regarded as the gold standard for diagnosing CD for many years. In the past decade, the European Society for Paediatric Gastroenterology, Hepatology and Nutrition (ESPGHAN) proposed the no-biopsy diagnostic approach under specific criteria (elevated tissue transglutaminase antibodies [TGA] > 10 × upper level of normal [ULN] and positive anti-endomysial antibodies [EMA] in a secondary blood sample) [1, 3]. Despite the potential advantages, concerns about missed pathologies and applicability in diverse populations have impeded the universal adoption of these guidelines [4]. Notably, the North American Society for Pediatric Gastroenterology, Hepatology and Nutrition (NASPGHAN) continues to recommend intestinal biopsy for confirming CD diagnosis in all cases, irrespective of TGA levels, since there is no standardisation of serological tests in the Unites States of America (USA) [5]. On the other hand, European studies clearly confirmed the reliability of serological tests in the no-biopsy approach [6, 7].

While ESPGHAN guidelines have reached acceptance in various regions over the years [8–11], guidelines for adults still emphasize the importance of biopsy confirmation for CD diagnosis [12, 13]. However, even for the adult population, ESPGHAN guidelines are gaining recognition and integration into diagnostic procedures, indicating a potential shift in the approach to diagnosing coeliac disease [13–16].

The aim of our study was to assess the diagnostic approach in children diagnosed with coeliac disease in Slovenia, and to calculate diagnostic delays in newly diagnosed symptomatic children.

## Methods

The study was carried out between March 2021 and October 2023 as a part of the CD SKILLS project (DTP 571), co-financed by the European Union Danube Transnational Programme.

### Participants and study design

For collection of patient data, a dedicated web-based form was designed within the scope of the Focus IN CD project (https://www.interreg-central.eu/Content.Node/surveys.html). This form underwent adaptation and translation into the Slovenian language. It encompassed inquiries regarding the coeliac disease diagnosis and management of newly diagnosed patients. Conducted as a prospective study, paediatric gastroenterologists across all hospitals in Slovenia responsible for managing children and adolescents with coeliac disease were invited to complete the questionnaire using medical documentation of children under 19 years who were diagnosed with CD between March 2021 and October 2023. The complete anonymization of reported data was ensured.

We analysed medical records of all included CD patients, focusing on levels of TGA at the time of diagnosis and determined whether the diagnosis was confirmed through a duodenal biopsy showing Marsh 2–3 lesions or through a no-biopsy approach. Additionally, we assessed the adherence to guidelines and calculated diagnostic delays, defined as the duration from the onset of first symptoms to confirmation of the diagnosis. We compared the results of Slovenian patients with the results of a similar study conducted in Central Europe within the Focus IN CD project in 2016.

### Statistical analysis

Statistical analysis was performed using IBM SPSS Statistics 24.0 (IBM Corp., Armonk, NY, USA). Descriptive statistics, one-way analysis of variance (ANOVA), independent samples t‑test and chi-square test were used for the analysis.

The study was approved by the National Medical Ethics Committee of the Republic of Slovenia (0120-12/2021/9).

## Results

Data from 160 children and adolescents (median age 8 years; min 7 months, max 18.5 years; 61.9% female) from five regional hospitals and two University Medical Centres in Slovenia were available for the analysis.

Twenty-seven children (16.9%) were asymptomatic at confirmation of the diagnosis (median age 9.5 years; min 2.5 years, max 18.5 years; 63% female) and were mostly diagnosed because of high-risk group screening (*N* = 25; 92.6%). The majority had a first-degree family member with CD (70.8%) and 20.8% had type‑1 diabetes mellitus. Other children were diagnosed with CD due to their signs and symptoms (median age 7 years; min 7 months, max 18 years; 61.7% female). Median diagnostic delay from first symptoms to confirmation of diagnosis was 6 months (min 0 months, max 7 years). There was no difference in diagnostic delays between symptomatic children diagnosed using biopsy and no-biopsy approaches (both 6 months).

The diagnostic procedure analysis revealed that 65% (*N* = 104) of included children (81.7% symptomatic) were diagnosed using a no-biopsy approach (median age 7 years). Among them, 97.1% (*N* = 101) of children had two separate serology samples, conforming to ESPGHAN 2020 guidelines. The remaining three children were diagnosed based on a single serological test and exhibited TGA > 10 × ULN and positive IgA EMA in the same blood sample.

Of the 56 children (86.7% symptomatic; median age 9 years; 91% Marsh–Oberhuber 2 or 3; 9% histological classification not used) who underwent duodenal biopsy, a further 10 children (17.8%) had TGA > 10 × ULN, making them eligible for the no-biopsy approach (Table 1). In these ten children, biopsies were predominantly conducted because parents insisted on the procedure (50.0%) or upper endoscopy was initially planned for other indications (40%). In a single case, biopsy was performed due to established institutional practice. Reasons for performing duodenal biopsies are presented in Table 2. None of the patients with TGA < 10 × ULN was diagnosed without duodenal biopsy.

**Table 1:** Diagnostic approach and tissue transglutaminase antibody (TGA) levels

		*Duodenal biopsy (N* *=* *56)*	*No-biopsy approach (N* *=* *104)*	
			*Single sample serology (N* *=* *3)*	*Two samples serology (N* *=* *101)*
*TGA level*	> 10 ×ULN	10	3	101^a^
	< 10 ×ULN	41	–	
	Not done/unable to clarify	5		

*TGA* tissue transglutaminase antibody, *ULN* upper limit of normal

^a^All criteria for no-biopsy approach fulfilled (including positive confirmatory antiendomysial antibodies (EMA)) [1]

**Table 2:** Reasons for performing duodenal biopsy in children with coeliac disease

Reason for duodenal biopsy	Number of children (%)
TGA < 10 × ULN	38 (67.9)
Selective IgA deficiency, not eligible for no-biopsy approach	4 (7.1)
Parents insisted on performing upper endoscopy with biopsy	5 (8.9)
Upper endoscopy was performed for other indications	6 (10.7)
Duodenal biopsy is an established institutional practice	3 (5.4)

*TGA* tissue transglutaminase antibody, *ULN* upper limit of normal

Genetic tests were conducted in 39.4% of included children, with 92% testing positive for DQ2 and 17% for DQ8 (6.3%), despite guidelines not deeming it necessary. Among children diagnosed using duodenal biopsy, 50% underwent genetic testing, while only 30.8% of those diagnosed using a no-biopsy approach underwent genetic testing (*p* < 0.05).

Comparing with 2016 data from the Focus IN CD project, Slovenia has seen a significant increase in use of the no-biopsy approach in recent years (Table 3; *p* = 0.001). The percentage of children who would have been eligible for the no-biopsy approach and were diagnosed with duodenal biopsy has decreased significantly (39.3% in 2016 vs. 17.8%; *p* < 0.05). Also, median diagnostic delays have shortened compared to 2016 (6 months vs. 7 months; *p* < 0.05).

**Table 3:** Comparison of diagnostic approach in Slovenia from 2016 and 2021–2023

	Slovenia 2016 (Focus IN CD;* N* = 45) (%)	Slovenia 2021–2023 (CD SKILLS;* N* = 160) (%)	Significance
No-biopsy approach	17 (37.8)	104 (65)	*p* = 0.001
Duodenal biopsy (% of all patients)	28 (62.2)	56 (35)	
*TGA level (% within group)*			
> 10 × ULN	11 (39.3)	10 (17.8)	*p* = 0.034
< 10 × ULN	15 (53.6)	41 (73.2)	
Not done/unable to clarify	2 (7.1)	5 (8.9)	–

*TGA* tissue transglutaminase antibody, *ULN* upper limit of normal

## Discussion

In the past decade, ESPGHAN guidelines for CD diagnosis have allowed the use of a no-biopsy approach in children and adolescents, provided specific criteria are met [1]. Since its implementation, this approach has undergone multiple assessments and has proven to be safe, including a large prospective international study involving 707 paediatric patients, where the no-biopsy approach demonstrated a positive predictive value for enteropathy of over 99% [6]. Subsequent to its initial inclusion in the ESPGHAN guidelines in 2012 [3], several studies in the following years have explored the application of the no-biopsy approach in children with coeliac disease [6–10]. Our data indicate a widespread adoption of the no-biopsy approach in Slovenia, with 65% of included children being diagnosed using this method. Interestingly, only 10% of children diagnosed via duodenal biopsy would still be eligible for the no-biopsy approach. Importantly, duodenal biopsies were performed for other valuable reasons based on available data. Notably, the use of the no-biopsy approach has increased significantly compared to our previous study in selected countries of Central Europe in 2016, where only 20% of children were diagnosed using this approach, and an additional 51.9% of biopsied children would have been eligible for the approach without the biopsy. Remarkably, even then, Slovenia emerged as the country most frequently employing the no-biopsy approach [17]. As in the study in Central Europe, there were no differences in diagnostic delays with regard to the diagnostic approach. The ESPGHAN guidelines were also adopted in most European countries, including Austria, Switzerland and Germany, where national guidelines are based on existing ESPGHAN guidelines [18].

In recent years, there has been notable exploration of the no-biopsy approach for diagnosing coeliac disease in adult populations [14–16]. Initial concerns about omitting biopsy, particularly in adults, due to potential risks of missing significant concomitant conditions including malignancy, have been addressed by recent studies from Italy and England [16, 19–22]. Interestingly, recent guidelines from the American College for Gastroenterology have proposed the use of a no-biopsy approach for diagnosing coeliac disease in children under specific circumstances [13]. However, it is important to note that this approach is not yet incorporated into the NASPGHAN guidelines for paediatric CD diagnosis [5].

Harmonization of guidelines is crucial, especially when considering the transition of care from childhood to adolescence and adulthood. This period can raise doubts about the accuracy of the original coeliac disease diagnosis, both for the patient and the new healthcare provider. Nevertheless, if the initial diagnosis in childhood was based on the ESPGHAN guidelines, there should be no uncertainty regarding the diagnosis [4].

The diverse clinical presentation of coeliac disease contributes significantly to diagnostic delays, especially in adults [23]. Although children generally experience shorter delays, some regions still report unacceptably prolonged durations. In our study, the median diagnostic delay was 6 months, aligning with the findings of our earlier research on diagnostic delays in children in Central Europe in 2016 [24]. However, regarding Slovenian patients in both studies, diagnostic delays have shortened significantly, which might be the consequence of various awareness-raising campaigns in past years in Slovenia. Another paediatric study on diagnostic delays in coeliac disease was conducted in New Zealand in 2019 by Coppell et al. [25]. In their study, the median diagnostic delay was significantly longer compared to our findings, reaching 1.5 years. Kosovo stands out with a mean delay in diagnosing CD in children at 2.2 years, notably longer than in other recent paediatric studies [26]. This prolonged delay may be attributed to the non-recognition of children with nonclassical clinical presentations [26].

Recent research provides a diverse picture of diagnostic delays in adult CD patients. A study in Denmark by Kårhus et al. [27] reported long self-reported delays in adult CD patients, with a mean delay of 5.8 years. Lenti et al. [28] found a much shorter median delay of 8 months in Italy, while Mansueto et al. [29] reported a mean delay of 9 years. Notably, Mehta et al. [30] reported a median diagnostic delay of 27 months in their study, revealing a correlation between the severity of celiac disease and the duration of diagnostic delay. It is important to note that most of these studies were based on patient recall and not on health records and, contrary to ours, were retrospective.

In conclusion, diverse guidelines exist globally for diagnosing coeliac disease, reflecting regional variations in diagnostic pathways despite the uniform nature of the disease. However, recent years have seen a promising trend towards harmonization, particularly in children and adolescents, with ESPGHAN guidelines gaining acceptance in many regions. Nevertheless, NASPGHAN guidelines for paediatric coeliac disease currently do not endorse the no-biopsy diagnosis. In the realm of adult coeliac disease diagnosis, there is a growing acceptance of the no-biopsy approach, although its full implementation is pending. Notably, Finland has already incorporated the no-biopsy approach into its national guidelines for diagnosing coeliac disease in adults [31]. The gradual integration of ESPGHAN guidelines into diagnostic procedures is underway, potentially influencing both paediatric and adult coeliac disease diagnoses. Also, the recurring theme of delayed recognition of CD underscores the need for improved awareness and timely diagnosis.
